# Pulmonary hypertension in bronchopulmonary dysplasia

**DOI:** 10.1038/s41390-020-0993-4

**Published:** 2020-06-10

**Authors:** Georg Hansmann, Hannes Sallmon, Charles C. Roehr, Stella Kourembanas, Eric D. Austin, Martin Koestenberger, for the European Pediatric Pulmonary Vascular Disease Network (EPPVDN)

**Affiliations:** 1 Department of Pediatric Cardiology and Critical Care, Hannover Medical School, Hannover, Germany; 2 Department of Pediatric Cardiology, Charité University Medical Center, Berlin, Germany; 3 Newborn Services, John Radcliffe Hospital, Oxford University Hospitals NHS Foundation Trust, Oxford, UK; 4 National Perinatal Epidemiology Unit, Nuffield Department of Population Health, Medical Sciences Division, University of Oxford, Oxford, UK; 5 Division of Newborn Medicine, Boston Children’s Hospital, Harvard Medical School, Boston, MA USA; 6 Division of Pediatric Pulmonary Medicine, Vanderbilt University, Nashville, TN USA; 7 Division of Pediatric Cardiology, Medical University of Graz, Graz, Austria

## Abstract

**Abstract:**

Bronchopulmonary dysplasia (BPD) is a major complication in prematurely born infants. Pulmonary hypertension (PH) associated with BPD (BPD-PH) is characterized by alveolar diffusion impairment, abnormal vascular remodeling, and rarefication of pulmonary vessels (vascular growth arrest), which lead to increased pulmonary vascular resistance and right heart failure. About 25% of infants with moderate to severe BPD develop BPD-PH that is associated with high morbidity and mortality. The recent evolution of broader PH-targeted pharmacotherapy in adults has opened up new treatment options for infants with BPD-PH. Sildenafil became the mainstay of contemporary BPD-PH therapy. Additional medications, such as endothelin receptor antagonists and prostacyclin analogs/mimetics, are increasingly being investigated in infants with PH. However, pediatric data from prospective or randomized controlled trials are still sparse. We discuss comprehensive diagnostic and therapeutic strategies for BPD-PH and briefly review the relevant differential diagnoses of parenchymal and interstitial developmental lung diseases. In addition, we provide a practical framework for the management of children with BPD-PH, incorporating the modified definition and classification of pediatric PH from the 2018 World Symposium on Pulmonary Hypertension, and the 2019 EPPVDN consensus recommendations on established and newly developed therapeutic strategies. Finally, current gaps of knowledge and future research directions are discussed.

**Impact:**

- PH in BPD substantially increases mortality. Treatment of BPD-PH should be conducted by an interdisciplinary team and follow our new treatment algorithm while still kept tailored to the individual patient.
- We discuss recent developments in BPD-PH, make recommendations on diagnosis, monitoring and treatment of PH in BPD, and address current gaps of knowledge and potential research directions.
- We provide a practical framework, including a new treatment algorithm, for the management of children with BPD-PH, incorporating the modified definition and classification of pediatric PH (2018 WSPH) and the 2019 EPPVDN consensus recommendations on established and newly developed therapeutic strategies for BPD-PH.

## Introduction

Bronchopulmonary dysplasia (BPD) represents a major cardiopulmonary complication in survivors of premature birth. The incidence of BPD is on the rise despite/because of the advances in neonatal intensive care.^1^ Besides impaired proximal airway and bronchoalveolar development, BPD is often associated with pulmonary vascular disease (PVD) and secondary pulmonary hypertension (PH). PH associated with BPD (BPD-PH) is characterized by abnormal vascular remodeling and rarefication of the pulmonary vasculature (vascular growth arrest),^2^ leading to increased pulmonary vascular resistance (PVR) and right heart failure. About 25% of infants with moderate to severe BPD develop PH^3,4^ that affects heart and lungs, greatly increasing mortality (47% of BPD infants die 2 years after diagnosis of PH).^5–7^

Compared to BPD without PH, BPD-PH is associated with suboptimal somatic growth and neurodevelopmental outcome,^8,9^ and higher rates of tracheostomy, increased use of supplemental oxygen, feeding problems, and frequent hospital admissions.^10^ Young adults born preterm (very low birth weight ≤1500 g; average gestational age 28 weeks) are at increased risk for PVD, PH, and right ventricular (RV) dysfunction.^11^

The increased survival of even the most immature infants makes infants with BPD-PH an ever-growing population among pediatric PH patients, requiring highly specialized care. Nevertheless, there is still a paucity of published data supporting the current management of children with BPD-PH.

In this article, we briefly review evolving concepts on the pathobiology of BPD-PH. We further discuss comprehensive diagnostic strategies, including the differential diagnosis of rather rare parenchymal and interstitial developmental lung diseases associated with PH. We provide a practical framework for the clinical management of children with BPD-PH, including established and newly developed therapeutic strategies, based on the 2018 World Symposium on Pulmonary Hypertension, and the 2019 updated consensus recommendations of the European Pediatric Pulmonary Vascular Disease Network (EPPVDN).^12,13^ Finally, we discuss current gaps of knowledge and potential future research directions.

## Definition and classifications of pediatric PH

The WSPH 2018 modified the definition and classification of PH presented in the “2015 ESC/ERS Guidelines.”^13,14^ Specifically, the lower limit of normal mean pulmonary arterial pressure (mPAP) was decreased from 24 to 20 mmHg.^15^ In adults, even mildly elevated mPAP values (20–24 mmHg, prognostic threshold 17 mmHg) were found to be independent predictors of poor survival.^16^ For consistency, this new definition of PH was also used in the pediatric WSPH 2018 document^13^ and the current 2019 EPPVDN guidelines,^12^ although the cut-off mPAP >20 mmHg remains arbitrary for children (Table 1). The Pulmonary Vascular Research Institute’s (PVRI) Panama Classification divided pulmonary hypertensive vascular disease (PHVD = PVD + PH) into 10 main categories, including BPD-PH, and more than 100 subcategories (2011).^17^ It should be noted that due to the physiologic postnatal elevation and subsequent decline of PVR after birth, the new mPAP cut-off value of 20 mmHg only applies to infants beyond 3 months of age. However, the aforementioned definition of PH does not specify whether chronological or corrected age should be used in preterm infants. Furthermore, prognostic studies on the relevance of borderline-elevated mPAP in older infants are currently lacking. Due to this ambiguity, BPD patients at risk for or with confirmed PH must be evaluated and cared for by a multidisciplinary team of PH specialists, including neonatologists, pediatric cardiologists, and pulmonologists, with experience in the care of infants with PH.^12,18,19^

**Table 1:** Developmental lung diseases associated with pulmonary hypertension.

Developmental defect	Vascular pathology	Diagnosis and treatment
Alveolar capillary dysplasia (ACD) with or without misalignments of veins (MPV)	Reduction of alveolar capillaries, thickening of alveolar septal tissue, and failure of the capillaries to make contact with the alveolar epithelium. Familial cases occur. Genomic and genetic deletions of the FOX transcription factor gene cluster at 16p24.1 and inactivating point mutations of FOXF1 were identified	Usually diagnosed post mortem by histological examination. Positive family history? Genetic testing. Symptomatic treatment to decrease PVR (O_2_, iNO, ECMO), bilateral lung transplant
Bronchopulmonary dysplasia (BPD)	Pre- and postnatal impact of exogenous risk factors on a structural and functional immature lung lead to postnatal impairment of angiogenesis and alveolarization associated with abnormal vascular function (increased tone, altered reactivity, impaired metabolism) and structure (smooth muscle cell proliferation, altered extra cellular matrix structure)	Defined by criteria based on supplemental oxygen at 36 weeks postmenstrual age. Typical chest X-ray findings. Supportive treatment including respiratory support, O_2_, corticosteroids, in case of PH sildenafil, diuretics (hydrochlothiazide, spironolactone), and ERA might be considered
Congenital diaphragmatic hernia (CDH)	Developmental defect leading to severe vascular remodeling and rarefication of the vascular bed. The defect is associated with variable degrees of lung hypoplasia	Clinical and radiological diagnosis. Surgical repair. Supportive treatment involves ventilation with permissive hypercapnia, HFOV, surfactant, pre- or postnatal glucocorticoids without clear benefit. Early repair on ECMO might be beneficial
Lung hypoplasia (primary and secondary)	Genetic abnormalities, severe reduction in amniotic fluid leading to reduced prenatal alveolar and vascular development. Secondary to congenital pulmonary malformations (CPAM, etc.)	Diagnosis by HR-CT depending on underlying cause. Supportive treatment (consider mechanical ventilation including HFOV, iNO, and surfactant). PH-specific medications often with only minimal effect
Pulmonary interstitial glycogenosis (PIG)	Rare non-lethal pediatric form of interstitial lung disease, possible male predominance. Infants present with respiratory distress	Diffuse interstitial infiltrates on chest radiography. Lung biopsy with histological cytoplasmic accumulation of glycogen in interstitial cells and associated lung growth abnormalities. Corticosteroids have been used with various efficacies. Spontaneous regression in some cases
Pulmonary alveolar proteinosis (PAP)	Rare lung disease in which abnormal accumulation of surfactant occurs within the alveoli, interfering with gas exchange and affecting lung growth. Possible cause anti-GM-CSF autoantibodies. GM-CSF receptor mutations in hereditary PAP	PAS-positive dense bodies in the distal airways on lung biopsy. Supportive treatment including repeated bronchioalveolar lavage and lung transplantation. GM-CSF might be considered in autoimmune PAP. Transplantation of macrophage progenitors in hereditary PAP (experimental)
Pulmonary lymphangiectasia	Rare developmental pulmonary disorder characterized by pulmonary subpleural, interlobar, perivascular and peribronchial lymphatic dilatation	Pleural (chylous) effusions with or without generalized edema. Radiologic progression of generalized hazy infiltrates in the neonatal period to a more perihilar interstitial pattern with hyperinflation (X-ray, CT, MRI). Lymphangiectasia on lung biopsy (periartierial, subpleural, interlobar). Supportive and symptomatic treatment (respiratory support, drainage). MCT nutrition. Octreotide and antiplasmin (experimental)
Surfactant protein (SP) abnormalities (SP-B and SP-C deficiency, ATP-binding cassette A3 mutation, thyroid transcription factor 1/Nkx2.1 homeobox mutation)	Genetic inheritance of surfactant deficiency leading to impaired lung development	Diagnosis by genetic testing. Might present as interstitial lung disease on chest imaging. Surfactant replacement has only temporary effect, lung transplantation. Supportive treatment. Corticosteroids might show some effect. Hydroxychloroquine (experimental, SP-C deficiency)

## Current concepts of the pathobiology of BPD-PH

PH associated with BPD mainly develops in survivors of extreme preterm birth as a result of incomplete lung development (abnormal alveolarization), postnatal hyperoxia-/hypoxia-triggered vascular remodeling, and the rarefication of pulmonary blood vessels (vascular growth arrest).^2^ Dysbalanced TGFβ/BMP^20^ and VEGF signaling in BPD are pathobiological hallmarks of BPD-PH.^2^ Risk factors for BPD include placental anomalies, extreme prematurity, very low birth weight, intra-uterine growth restriction, perinatal infections, oxygen toxicity, and mechanical ventilation.^2,21^ While the precise mechanisms of PH associated with BPD are still unknown, it is likely they involve insults such as inflammation and endothelial dysfunction, perpetuated through alveolar hypoxia.^2,21^ In older infants, a cycle of progressive respiratory insufficiency, hypoxia, and compromised pulmonary perfusion aggravates PH, contributing to the increased mortality observed in BPD-PH infants.

## Diagnosis of BPD and BPD-PH

Clinical suspicion of BPD-PH should be raised in every infant with a history of prematurity who requires supplemental oxygen and/or invasive or non-invasive respiratory support. Traditionally, BPD is defined clinically as supplemental oxygen use either at a corrected age of 36 gestational weeks (p.m.) or after 28 days of postnatal life in infants born <32 weeks of gestational age.^22^ However, this definition has recently been challenged and a new definition of BPD was proposed that includes infants on any form of respiratory support (such as high-flow nasal cannula) at 36 corrected weeks (p.m.), even in the absence of supplemental oxygen use.^23^ The significance of this new, alternative BPD definition for the frequency and morbidity of BPD-PH still needs to be investigated, and has not been used yet for this review article. In any case, PH/PHVD might occur before the formal diagnosis of BPD can be made. Indeed, echocardiographic screening may help identify infants at risk for BPD-PH as early as 7 days of life.^4^

**Fig. 1:**
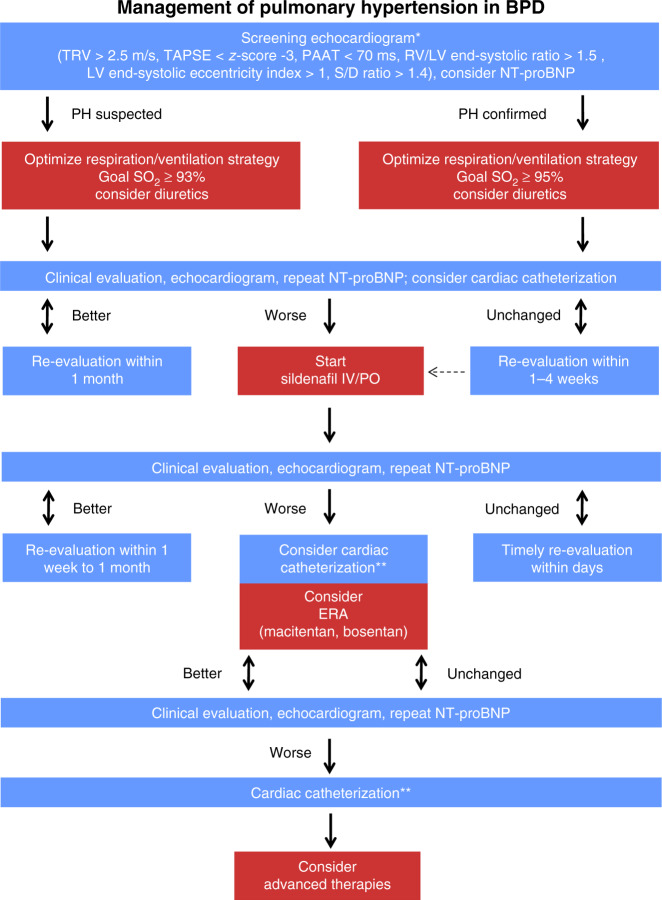
Management of pulmonary hypertension in bronchopulmonary dysplasia. BPD—bronchopulmonary dysplasia; ERA—endothelin receptor antagonist; iNO—inhaled nitric oxide; LV—left ventricle; RV—right ventricle; PAAT—pulmonary artery acceleration time; PH—pulmonary hypertension; S/D ratio—systolic to diastolic duration ratio; TAPSE—tricuspid annular posterior systolic excursion; TRV—tricuspid regurgitation velocity. *Screening for pulmonary hypertension by transthoracic echocardiography is indicated (1) if severe respiratory compromise is present in any preterm infant <28 weeks of gestation, (2) in any infant with established BPD at corrected age of 36 weeks gestation (p.m.) and before discharge, (3) in any infant with prolonged oxygen requirement, poor growth, and unsatisfactory clinical improvement. **Cardiac catheterization should be considered before (i) introduction of a second PH-specific medication and is advised if PH worsens under dual therapy or if unsatisfactory treatment response is seen in any infant with PH at a corrected postnatal age of >3 months.

While BPD-specific changes are detected on conventional chest X-ray, the initial assessment of suspected PH is based on transthoracic echocardiography (TTE) (Fig. 1). Here, one should not rely on a single TTE parameter, but follow a multiparametric approach. Even with a history of immaturity, certain (rare) interstitial or parenchymal developmental lung diseases that can mimic BPD/BPD-PH must be included in the differential diagnosis (Table 1). Cardiac anomalies should be ruled out as these might affect therapy: for example, the presence of hemodynamically significant cardiovascular post-tricuspid shunts, such as large ventricular septal defects, often preclude the use of pulmonary vasodilators due to an aggravation of the shunt volume resulting from decreased PVR.^12^

### Echocardiography for diagnosis and monitoring of PH

TTE is an important diagnostic and monitoring modality.^12,24–27^ Screening for PH by TTE is indicated (1) if severe respiratory compromise is present in any preterm infant <28 weeks of gestation, (2) in any infant with established BPD at corrected age of 36 weeks gestation (p.m.) and before discharge, (3) in any infant with prolonged oxygen requirement, poor growth, unsatisfactory clinical improvement,^12^ and has also been suggested at 7 days of life to identify infants at risk for BPD-PH.^4^ Comprehensive TTE allows for complete initial assessment of cardiovascular anatomy and may also confirm RV pressure elevation through the measurement of the maximal velocity of the tricuspid regurgitation (TR) jet by continuous wave Doppler; TTE can detect impaired RV systolic function through qualitative assessment in several views, and quantitatively by means of a reduced age-related tricuspid annular plane systolic excursion as surrogate of longitudinal systolic RV function.^12^ Of note, TTE variables have different age-related reference ranges and variable impact on the accuracy of the diagnosis “PH” in children vs. adults, especially in young infants.^24^ The presence of a hemodynamically significant post-tricuspid shunt makes TTE estimation of PAP elevation less accurate.^28^

The relevant non-invasive imaging risk variables currently evaluated for infants and young children with PH,^12^ including those with BPD-PH, are listed in Table 2.

**Table 2:** Echocardiographic determinants of PH risk in infants.

Lower risk	Determinants of risk	Higher risk
Minimal RA/RV enlargement No RV systolic dysfunction RV/LV ratio <1 (PSAX) TAPSE normal (*z* >−2) S/D ratio <1.0 (TR jet) PAAT >100 ms (>1 year old)	Echocardiography	Severe RA/RV enlargement RV systolic dysfunction RV/LV ratio >1.5 (PSAX) TAPSE ↓↓ (*z* <−3) S/D ratio >1.4 (TR jet) PAAT <70 ms (>1 year old) Pericardial effusion

TTE measurement	To estimate	Comment
TRV (m/s)	RVSP	Depends on the angle of continuous wave Doppler interrogation^71^ (full Doppler envelopes)
S/D ratio	PAP	Requires presence of well-defined TR^71^ (full Doppler envelopes)
PRV (m/s), diastolic max.	Mean PAP	Requires presence of well-defined PR^71^ (full Doppler envelopes)
PRV (m/s), diastolic min.	Diastolic PAP	Requires presence of well-defined PR^71^ (full Doppler envelopes)
PAAT (ms)	PAP, PVR	Measured in PSAX. PAAT was suggested to be an adequate follow-up parameter for assessing BPD-PH^72,73^ despite the notion that the normal reference range for PAAT in first year of life is particularly broad (54–116 ms).^74^ Heart rate dependent
RVOT VTI (cm)	PAP, PVR	Depends on the angle of PW Doppler interrogation. Increased TRV/RVOT VTI ratio in PH^75^
RA area (cm^2^), end systole	RA dilation	Requires standard, on-axis four-chamber imaging^25^
RA/LA ratio, end systole	RA dilation	Requires standard, on-axis four-chamber imaging
RV/LV ratio, end systole	RV dilation	Requires standard, on-axis PSAX imaging^76^
RVES RI	RV remodeling	Useful especially in the absence of reliable TR and PR jets^77^
LV eccentricity index, end systole	RV end-systolic pressure vs. LV end-systolic pressure; LV filling and compression	Requires standard, on-axis PSAX imaging^78^
TAPSE	Systolic longitudinal RV function	Not a surrogate of segmental or radial changes in RV function^71^

*LV* left ventricle, *RA* right atrium, *RV* right ventricle, *PAAT* pulmonary artery acceleration time, *PH* pulmonary hypertension, *PR* pulmonary regurgitation, *PRV* pulmonary artery regurgitation velocity in diastole, *PSAX* parasternal short axis view, *PVR* pulmonary vascular resistance, *RVES RI* right ventricular end-systolic remodeling index, *RVSP* right ventricular systolic pressure, *S/D* ratio systolic to diastolic duration ratio, *TAPSE* tricuspid annular posterior systolic excursion, *TTE* transthoracic echocardiography, *TR* tricuspid regurgitation, *TRV* tricuspid regurgitation velocity, *Z* *z*-score.

### Magnetic resonance imaging and chest computed tomography

Magnetic resonance imaging (MRI) and computed tomography (CT) have become essential non-invasive imaging modalities in suspected or confirmed PH.^12^ MRI offers the ability to assess pulmonary and systemic blood flow, pulmonary regional perfusion, PA and aortic diameters, (bi-) ventricular cardiac function, and myocardial tissue characteristics.^29^ More recently, lung MRI (e.g., TWIST, time-resolved angiography with stochastic trajectories) was introduced to assess lung perfusion in PHVD.^30,31^ A recent study on MRI in BPD-PH revealed that the pulmonary artery to aorta ratio (PA/AO) increased with BPD severity. An increased PA/AO and MR-derived LV end-systolic eccentricity index (MR-LVEI) were associated with increased length of stay and duration of respiratory support in BPD infants.^31^ Both increased PA/AO ratio and MR-LVEI were associated with PH therapy during hospitalization and at discharge. The primary role of chest CT is to detect lung parenchymal disorders, thromboembolic disease, and vascular abnormalities, such as pulmonary vein stenosis, which may be causally linked to evident PH. The key role of chest imaging, including newer MRI techniques in differentiating interstitial and parenchymal developmental lung disease has been discussed elsewhere.^32^ The significant risk of sedation/general anesthesia in PH patients need to be balanced against the potential gain of information of MRI or CT studies, and their impact on the future therapy of the individual PH patient.^12^

### Diagnostic cardiac catheterization in suspected or confirmed BPD-PH

Cardiac catheterization is rarely necessary to make the diagnosis of BPD-PH early on. Although cardiac catheterization with acute pulmonary vasoreactivity testing (AVT) generally is recommended in pediatric PH prior to the initiation of pulmonary vasodilator therapy,^11–13^ exceptions may be made when the risk of the cardiac catheterization outweighs the potential benefits; for instance, severe hemodynamic instability (premature infants) or evidence of systemic vasculopathies. However, cardiac catheterization for the diagnosis and assessment of BPD-PH should be considered before introduction of a second PH-targeted medication; cardiac catheterization is advised if PH worsens under dual PH-targeted therapy or if an unsatisfactory response to PH-targeted pharmacotherapy is seen in any infant with PH at a corrected postnatal age of >3 months (Fig. 1).^13^ Additional indications include concern for vascular abnormalities (e.g., pulmonary vein stenosis),^33^ other features of an atypical response to therapy, and/or failure to thrive.^34^

A systematic cardiac catheterization protocol for pediatric PH is available.^12^ However, the complexity of pediatric PH/PVD often requires individualized adaptations of these recommendations. A recent study found that AVT may aid in the assessment of disease severity and management of BPD-PH. In this cohort of 26 infants with BPD, 35% had positive AVT, which was associated with better long-term outcomes. AVT also distinguished higher from lower risk PH in infants with BPD better than baseline pulmonary hemodynamics.^35^ Others found that the severity of lung disease as assessed by impaired oxygenation at cardiac catheterization did not correlate with mortality while the presence of pulmonary vein stenosis was associated with death.^33^

### Biomarkers

Blood biomarker concentrations contribute to tailoring longitudinal medical care of PH patients.^12^ Brain natriuretic peptide (BNP) and its N-terminal cleavage product (NT-proBNP) are secreted by cardiomyocytes in response to ventricular wall stress due to pressure overload and/or volume expansion. Both biomarkers exhibit similar relative stress-induced release and age dependency, but NT-proBNP has the longer half-life (118 vs. 18 min). In a meta-analysis of 25 small-scale pediatric studies, serum NT-proBNP was identified as significant prognostic factor in pediatric PH and suggested to aid in the longitudinal assessment of patients^36^ (reference values of NTproBNP-BNP^37^). Recent studies have also shown the applicability of serum NT-proBNP as a suitable biomarker in infants with BPD-PH.^38–40^ Based on our experience, especially in infants, absolute values of NT-proBNP have a high inter-individual variance, are strongly dependent on postnatal and gestational age, and thus have limited value in establishing the diagnosis or severity of PH, especially in young infants. However, longitudinal assessment of NT-proBNP in combination with echocardiography is very useful to assess disease progression and/or response to treatment.^41–44^

Meanwhile, additional biomarkers are under consideration and may contribute to future PH management.^12,40^

## Treatment

### Supportive measures

Supplemental oxygen should be supplied when target oxygen saturations are >93% for infants with suspected and >95% for infants with proven PH (Fig. 1).^12^ Maximizing non-invasive respiratory support as much as possible in order to avoid mechanical ventilation and therefore aggravation of lung injury is advisable. However, atelectasis causes an increased ventilation/perfusion mismatch and local hypoxic vasoconstriction, and thus needs to be avoided. In addition, treatment with diuretics, that is, hydrochlorothiazide and spironolactone may be considered in infants with severe BPD.^12^ However, diuretic therapy in preterm infants remains controversial, given its negative impact on growth and the risk for metabolic bone disease. Thus, indications and duration of diuretic treatment should be discussed in a multidisciplinary team, involving cardiologists, neonatologists, pulmonologists, and nutritionists. Infants with severe BPD have higher caloric requirements, which should be accounted for in their nutritional plans (up to 160 kcal/kg). Infants with BPD-PH are prone to a sudden elevation of PVR when undergoing stressful procedures (“PH crisis”) and often require sufficient sedation or even analgesia/relaxation before such procedures are performed in an intensive care setting. Administration of inhaled nitric oxide and phosphodiesterase-3 inhibitors, such as milrinone, which are frequently used for acute treatment of PH in infants (e.g., in PPHN or acute deterioration of BPD-PH), are beyond the scope of this review and discussed elsewhere.^12^

### Pharmacotherapy

#### General considerations

The overall goal of therapy for PH patients, adult or pediatric, is (1) to induce pulmonary arterial vasodilation, (2) to pressure unload and support the RV, (3) to avoid coronary ischemia and heart failure, (4) improve clinical outcomes and quality of life, and (5) to improve signs and symptoms, and thus the aggravation of PH.^12,14,45–47^ Of note, especially oral pulmonary vasodilator therapy may amplify ventilation and perfusion mismatch in any type of PH, especially in the setting of lung disease.

Regardless of a patient’s age or type of PH (group 1–5 PH),^15^ PH-targeted therapy currently focuses on three main molecular pathways: the nitric oxide pathway, the endothelin pathway, and the prostacyclin pathway (Table 3). While only two drugs have so far been approved by the regulatory European Medicines Agency (EMA) for pediatric patients with PAH, that is, sildenafil (body weight ≥8 kg and >1 year old) and bosentan (age >1 year), only bosentan has been approved by the Food and Drug Administration (FDA) for chronic use in PH children > 3 years of age. However, both sildenafil and bosentan are frequently used for acute and long-term treatment of infants with BPD-PH (Fig. 1).^41,42^ In the absence of randomized clinical trial data, use of PH-targeted medications in infants is based on expert opinion and experience, underlining the necessity of comprehensive evaluation in PH expert centers according to current international recommendations.^12,48^ The primary treatment goal for patients with PH associated with developmental lung diseases (group 3 PH)^15^ is to treat the underlying lung disease.^12,48^ However, BPD-PH patients are likely to benefit from vasodilator treatment.^12,48,49^ A recent, retrospective analysis showed clinical and echocardiographic improvements with a survival rate of 95% during a median follow-up time of 2 years in infants treated with PH-targeted therapy for BPD complicated by PH.^42^

**Table 3:** Pharmacotherapy of BPD-PH.

Drug	Mechanism	Comment
Sildenafil	PDE5 inhibitor	Most widely used in BPD-PH. Aggravates gastroesophageal reflux. EMA approved for children >1 year. The EPPVDN consensus (2019) recommends sildenafil at a maximum dose of 1 mg/kg/dose every 6 h in the first year of life, followed by the EMA drug recommendations for sildenafil: 10 mg every 8 h for patients ≥10 kg bodyweight and >12 months old, and 20 mg every 8 h for patients ≥20 kg bodyweight)
Tadalafil	PDE5 inhibitor	Alternative to sildenafil, less frequent dosing. No data on BPD-PH
Riociguat	Stimulator of soluble guanylatcyclase (sGC)	“Dual” mode of action (sGC activator and stimulator). No data on BPD-PH
Bosentan	ERA	ET_1A_- and ET_1B_-receptor antagonist. Most widely used in BPD-PH in combination with PDE-5 inhibitor. May increase liver transaminases. Lowers circulating sildenafil levels. EMA approved for children with PAH >1 year based on BREATHE-3 and FUTURE-1 trials FDA-approved for children with PAH >3 years Maximum target bosentan dose is 2 mg/kg body weight twice daily (4 mg/kg/day)
Macitentan	ERA	ET_1A_- und ET_1B_-receptor antagonist. No liver toxicity. Does not lower sildenafil levels. Only limited experience in BPD-PH
Ambrisentan	ERA	Selective ET_1A_-receptor inhibition. May increase liver transaminases. Does not lower sildenafil levels. No experience in BPD-PH
Iloprost	PCA	Some experience in infants with PPHN and CHD-PAH either in combination with sildenafil or as monotherapy. May cause airway hyperreactivity
Epoprostenol	PCA	No experience in BPD-PH
Trepostinil	PCA	No experience in BPD-PH
Selexipag	Oral IP-receptor agonist	Only few reports on pediatric use in PAH, and CHD-PAH. Offers potential of oral “triple”-combination therapy. See EPPVDN pediatric selexipag study (2020)^43^

*BPD-PH* bronchopulmonary dysplasia-associated pulmonary hypertension, *CHD* congenital heart disease, *EMA* European Medicines Agency, *ERA* endothelin receptor antagonist, *ET* endothelin, *IP* prostacyclin receptor, *PDE5* phosphodiesterase 5, *PAH* pulmonary arterial hypertension, *PCA* prostacyclin analog, *PPHN* persistent pulmonary hypertension of the newborn, *FDA* Food and Drug Administration.

#### Pharmacotherapy to modify the nitric oxide pathway

Phosphodiesterase-5 (PDE5) inhibitors are the most commonly employed drugs acting on the nitric oxide (NO) pathway, namely sildenafil and tadalafil. PDE5 inhibitors inhibit primarily PDE5 (NO/cGMP pathway), thus inducing vasodilation through vascular smooth muscle cell relaxation, and also exhibit anti-proliferative effects.^50^ STARTS-1 and its extension phase, STARTS-2,^51^ were the first pediatric randomized, placebo-controlled clinical trials in PAH. The STARTS study demonstrated improvement in secondary outcomes among those PH patients on medium- or high-dose sildenafil, including functional class and PVR. Controversially, the STARTS trial detected an increase in mortality among those subjects receiving high-dose sildenafil vs. placebo group, which in 2013 led to a warning of the EMA not to use higher doses, and rejection of approval by the FDA. Nevertheless, sildenafil is—by most centers and experts—regarded as a safe and efficacious first-of-choice drug for PH/PVD in children, and its widespread use and recommendation in formal guidelines continues.^12,42^ Sildenafil at standard dose should be considered in children with BPD-PH as improvements of clinical status and PAP have been demonstrated for oral sildenafil therapy.^41^ Tadalafil is reported to be widely used in North America due to its reduced dosing frequency, compared to sildenafil. Common to all PDE5 inhibitors, gastroesophageal reflux is an often reported unwanted effect that is also highly relevant to neonates and infants.^46^ Riociguat is a novel oral agent with dual mode of action that acts in synergy with endogenous NO and directly stimulates soluble guanylyl cyclase, with only very limited pediatric experience.^47^

#### Pharmacotherapy to modify the endothelin pathway

Bosentan, an oral non-selective endothelin A- and B-receptor antagonist (ERA), counteracts the vasoconstrictive and mitogenic effects of endothelin-1 in PAH patients. Elevated liver aminotransferases may occur with bosentan use as a serious adverse event, but seem to be less frequent in children under 12 years than in adults and children ≥12 years of age (2.7% vs. 7.8%).^52–55^ Nevertheless, monthly liver enzyme and function testing should be performed in children receiving bosentan.^12,14^ ERA treatment may also be considered in BPD infants.^12^ Macitentan provides an alternative to bosentan that shows a more favorable safety profile (less liver toxicity, no teratogenicity). Macitentan has been studied in a small prospective pediatric study and was shown to be safe and efficacious as add-on therapy.^44^ Off-label use of macitentan also has been reported in BPD-PH patients.^56^ Of note, bosentan lowers circulating sildenafil levels, while the other two ERA, ambrisentan and macitentan, do not.^12^

#### Pharmacotherapy to modify the prostacyclin pathway: prostacyclin and prostacyclin analogs (PGI_2_ and PGI_2_ analogs; PCA)

Prostacyclin (PGI_2_) and its analogs activate the PGI_2_receptor (IP receptor) and lead to vasodilation, inhibit vascular smooth muscle cell proliferation, and have anti-inflammatory effects. PCAs were developed which facilitate intravenous, but also subcutaneous, inhaled, and more recently oral delivery routes.^12^ As with other pulmonary vasodilators, PGI_2_ or PCAs may have off-target effects prompting side effects, including headache, jaw pain, nausea, vomiting, abdominal pain, diarrhea, and flushing. Inhaled PGI_2_ or PCAs may cause airway hyperreactivity,^57^ in addition to headaches and flushing. However, the use of especially inhaled PCAs has been reported in infants, for example, inhaled iloprost might be used as monotherapy or as adjuvant.^58^ *Selexipag* is a new oral selective prostacyclin receptor (IP receptor) agonist, but not yet systematically studied in “BPD-PH”. The first prospective, pediatric multicenter study on PAH-combination therapy indicates that selexipag can be used safely even in small children (4–10 kg bodyweight), and that a positive drug response can be expected in half of the children treated with selexipag.^43^

### Interventional and surgical procedures

In children with BPD, large left-to-right atrial or ventricular shunts are not well tolerated, and may be closed early if PVD is mild (i.e., no or only mild PVR elevation at cardiac catheterization).^59^ However, aorto-pulmonary connections (e.g., a patent ductus arteriosus, PDA) may facilitate decompressing a pressure-loaded RV in severe PH/PVD.^12^ Procedures for palliation of children with severe PH and RV failure include the creation of a right-to-left shunt with the aim to decompress the right heart and to increase cardiac output. These interventions decompress the right atrium only (balloon atrial septostomy, BAS) or the RV (reverse Potts shunt). While the interatrial shunt resulting from BAS only secures some cardiac output in critical supra-systemic PAP elevations, the “endogenous” reverse Potts shunt (e.g., through stenting of a small PDA) clearly decomprresses the RV and lowers RV pressure.

The resulting hemodynamic situation after both interventional procedures is similar to the physiology of Eisenmenger patients. In imminent RV failure, an existing PDA can be balloon-dilated and stented (endogenous Potts shunt), representing an elegant procedure to decompress a failing RV and may be considered in patients with supra-systemic PH refractory to any medical treatment, including combination PH-targeted therapy.^12,60^ Similarily, in acute deterioration, PGE1 may be used to keep the PDA open at the expense of hypoxemia in the lower half of the body.

## Prevention of BPD-PH

Two recent studies on clinical risk factors for BPD-PH identified these to be similar to the risk factors known to be associated with BPD: extreme prematurity, mechanical ventilation, tracheostomy, tracheitis, intraventricular hemorrhage (grade ≥3) and systemic steroid use, hyperoxia, and inflammation/sepsis.^61,62^ Thus, preventive measures to decrease BPD frequency and severity are of pivotal importance in order to lower the BPD-PH burden among preterm infants. These preventive measures include, but are not limited to: less use of invasive mechanical ventilation and predominant use of non-invasive respiratory support strategies, minimally invasive surfactant application, avoidance of hyper- and hypoxia, caffeine, corticosteroids, and reduction of infections.^63–66^ Further research is required to determine the role of caffeine and corticosteroids on the developing pulmonary vasculature. The same is true for a possible contribution of a hemodynamically significant PDA (here: sole left-to-right shut) to PH and PVD in BPD infants. In addition, early biomarkers, detection, and treatment of PH + PVD might hold the potential to decrease BPD-PH disease severity (Table 4).

**Table 4:** Future research directions in BPD-PH.

New discoveries in the pathobiology
Mechanistic studies on the pathobiology of BPD-PH and the role of common neonatal conditions such as respiratory support, surfactant, nutritional care, ductus arteriosus and inflammation on the developing pulmonary vasculature
Investigating mechanisms of right heart adaptation and remodeling in preterm infants with and without BPD
Identification of risk factors
Identification of pre- and postnatal risk factors relevant for the development of PHVD/PH in BPD infants
Identification of preventive measures against PHVD/PH during the fetal and early neonatal periods
Role of genetic risk factors in the pathogenesis of BPD-PH
Role of cardiac catheterization and advanced imaging (CT/CMR) in severe BPD-PH
Early detection
Clinical and echocardiographic parameters for early detection of PHVD/PH in preterm infants at risk for BPD
Innovative and efficient therapies
Research on underlying beneficial mechanisms, mode of delivery, and efficacy of stem-cell-based therapies, including cell-free preparation (e.g., conditioned media, exosome-based therapies)
Trials on the safety and efficacy of pulmonary vasodilatory therapy in preterm infants with BPD-PH
Potential role of vasodilatory combination therapy for BPD-PH
Potential role of new PAH-drugs, such as macitentan, selexipag, riociguat in BPD-PH
Clinical follow-up
Further research on biomarkers in BPD-PH (early detection and follow-up)
Definition of comprehensive multidisciplinary follow-up protocols, including neurodevelopmental aspects

*BPD-PH* bronchopulmonary dysplasia-associated pulmonary hypertension, *CT* computerized tomography, *CMR* cardiac magnetic resonance, *PHVD* pulmonary hypertensive vascular disease.

## Conclusions and future research directions

BPD-PH represents a significant burden to pediatric PH programs, but has been relatively understudied to date. The current wide ambiguity regarding optimal diagnosis, treatment, and monitoring of disease progression creates several high yield opportunities for study and practice improvement (Table 4). These include, but are not limited to: (1) comprehensive observational research cohort studies of the epidemiology of PH among high risk individuals with lung disease; (2) formal therapeutic trials incorporating PH-specific therapies in concert with traditional disease-specific approaches (e.g., oxygen and nutritional support); (3) novel therapies, including those directed at the underlying lung disease and those directed toward the pulmonary vasculature must be developed and studied, including modern oral combination pharmacotherapy; and, (4) studies designed to prevent and detect PH earlier among those with significant lung disease. Finally, cell-based therapies such as the use of mesenchymal stem cells (MSCs), MSC-conditioned media (cell media) or MSC extracellular vesicles/exosomes have shown promising preclinical results in reversing BPD-PH and several clinical trials are underway.^67–70^ A phase 1 study on the use of mesenchymal stem cell-derived extracellular vesicles in preterm neonates at high risk for BPD is underway (NCT03857841).
